# HPV testing in primary screening of older women

**DOI:** 10.1038/sj.bjc.6690730

**Published:** 1999-10

**Authors:** J Cuzick, E Beverley, L Ho, G Terry, H Sapper, I Mielzynska, A Lorincz, W-K Chan, T Krausz, P Soutter

**Affiliations:** Imperial Cancer Research Fund, 61 Lincoln's Inn Fields, London, WC2A 3PX, UK; University College London Medical School, London, UK; Acton Health Centre, London, UK; Digene Diagnostics Inc, Silverspring, MD, USA; 9 Greenfield Drive, London, UK; Hammersmith Hospital, Du Cane Road, London, UK

**Keywords:** hybrid capture, cancer screening, cervical cancer, human papillomaviruses

## Abstract

Certain types of the human papilloma virus (HPV) are well established as the primary cause of cervical cancer. Several studies have shown that HPV testing can improve the detection rate of high-grade cervical intraepithelial neoplasia (CIN), but these have been carried out primarily in younger women. In this study we evaluated the role of HPV testing as an adjunct to cytology in women aged 35 or over. An additional aim was to evaluate commercially available kits for HPV testing. A total of 2988 eligible women aged 34 or more attending for a routine smear in 40 general practitioner practices received HPV testing in addition to routine cytology, after having given written informed consent. Samples were assayed by polymerase chain reaction (PCR) and two versions of the Hybrid Capture test for HPV, and women were invited for colposcopy if there was any cytological abnormality (including borderline smears) or the PCR test was positive. Any apparent abnormality was biopsied and loop-excision was performed as necessary. CIN was judged by histology; 42 women had high-grade CIN, of which six were cytology negative (86% sensitivity for borderline or worse) and three had a borderline smear (79% sensitivity for mild dyskaryosis or worse). The positive predictive value of a borderline smear was only 3.1%. Eleven high-grade lesions were negative by the PCR HPV test (sensitivity 74%). The first generation Hybrid Capture II test had a similar sensitivity but an unacceptably high false positive rate (18.3%), while the newer Hybrid Capture II microtitre kit had a 95% sensitivity and a 2.3% positivity rate in normal women when used at a 2 pg ml^−1^ cut-off (positive predictive value 27%). Cytology performed very well in this older cohort of women. The newer Hybrid Capture II microtitre test may be a useful adjunct, especially if the results reported here are reproducible in other studies. A combined screening test offers the possibility of greater protection and/or longer screening intervals, which could reduce the overall cost of the screening programme. © 1999 Cancer Research Campaign


*British Journal of Cancer* (1999) **81**(3), 554–558
© 1999 Cancer Research Campaign
Article no. bjoc.1999.0730

### HPV testing in primary screening of older women

**J Cuzick[1], E Beverley[1], L Ho[2], G Terry[2], H Sapper[3], I Mielzynska[4], A Lorincz[4], W-K Chan[5], T Krausz[6] and P Soutter[6]**

[1]Imperial Cancer Research Fund, 61 Lincoln's Inn Fields, London WC2A 3PX, UK; [2]University College London Medical School, London, UK; [3]Acton Health Centre, London, UK; [4]Digene Diagnostics Inc., Silverspring, MD, USA; [5]9 Greenfield Drive, London, UK; [6]Hammersmith Hospital, Du Cane Road, London, UK

**Summary** Certain types of the human papilloma virus (HPV) are well established as the primary cause of cervical cancer. Several studies have shown that HPV testing can improve the detection rate of high-grade cervical intraepithelial neoplasia (CIN), but these have been carried out primarily in younger women. In this study we evaluated the role of HPV testing as an adjunct to cytology in women aged 35 or over. An additional aim was to evaluate commercially available kits for HPV testing. A total of 2988 eligible women aged 34 or more attending for a routine smear in 40 general practitioner practices received HPV testing in addition to routine cytology, after having given written informed consent. Samples were assayed by polymerase chain reaction (PCR) and two versions of the Hybrid Capture test for HPV, and women were invited for colposcopy if there was any cytological abnormality (including borderline smears) or the PCR test was positive. Any apparent abnormality was biopsied and loop-excision was performed as necessary. CIN was judged by histology; 42 women had high-grade CIN, of which six were cytology negative (86% sensitivity for borderline or worse) and three had a borderline smear (79% sensitivity for mild dyskaryosis or worse). The positive predictive value of a borderline smear was only 3.1%. Eleven high-grade lesions were negative by the PCR HPV test (sensitivity 74%). The first generation Hybrid Capture II test had a similar sensitivity but an unacceptably high false positive rate (18.3%), while the newer Hybrid Capture II microtitre kit had a 95% sensitivity and a 2.3% positivity rate in normal women when used at a 2 pg ml$^{-1}$ cut-off (positive predictive value 27%). Cytology performed very well in this older cohort of women. The newer Hybrid Capture II microtitre test may be a useful adjunct, especially if the results reported here are reproducible in other studies. A combined screening test offers the possibility of greater protection and/or longer screening intervals, which could reduce the overall cost of the screening programme.
© 1999 Cancer Research Campaign

**Keywords:** hybrid capture; cancer screening; cervical cancer; human papillomaviruses

In a previous study we found that testing for four high-risk human papilloma (HR-HPV) types almost doubled the detection rates of cervical intraepithelial neoplasia (CIN) 2/3 when added to cervical cytology in routine screening (Cuzick et al, 1995). In particular, 44% of these lesions occurred in women with negative cytology and another 23% had only mild or borderline cytological abnormalities. The smears were taken by experienced personnel and several reviews of the smears confirmed the findings. Similar results have now been reported in other studies (Cox et al, 1995; Hatch et al, 1995; Walboomers et al, 1995; Ferenczy et al, 1996).

A limitation of these studies is that most women were relatively young. In our previous study 87% were under 40 years of age. In general, HPV infection in younger women tends to be at a high viral load, but often spontaneously regresses, whereas in older women the virus is more prone to persist, but often the viral load is lower (Hildesheim et al, 1994; Ho et al, 1995). We now report a study in women aged 35 or over to evaluate the role of HR-HPV testing in primary screening of older women. An additional aim was to evaluate commercially available kits for HPV testing.

### PATIENTS AND METHODS

Women aged 35 years and over who were attending for a routine smear in 40 general practitioner (GP) practices were asked to join a study in which HPV testing would be performed in addition to routine cytology. Ethical approval was obtained from the referral hospital ethics committee (Hammersmith Hospital) and informed consent was obtained from all participants after a written and oral explanation of the study was provided by the practice nurse or GP.

A cervical smear was taken using an Aylesbury spatula and put onto a glass slide in the conventional manner. Remaining material on the spatula was transferred to narrow tubes by a cotton swab for polymerase chain reaction (PCR) analysis of high risk HPV types (16, 18, 31, 33, 35, 45, 51, 52, 56, 58) using consensus PCR (Ting and Manos, 1990) and the SHARP detection system (Digene Corp. (PCR/SHARP). A second sample was then obtained and placed in a standard sample transport medium to evaluate a simpler signal-amplified HPV test (Hybrid Capture, Digene Corp.). In the first half of the study this sample was collected with a dacron swab and analysed by the Hybrid Capture Tube assay (HC-I), which had a sensitivity of approximately 10 pg ml$^{-1}$. In the second half a conical cervical brush sampling device was used, and the samples were analysed by the newer Hybrid Capture microtitre format assay (HC-II). Positivity was evaluated at the conventional 1 pg/ml$^{-1}$ level as well as higher thresholds of 2 pg ml$^{-1}$ and 4 pg ml$^{-1}$.

For the PCR/SHARP assay, PCR amplification was carried out using consensus primers MY09/11 in a microtitre format and the presence of high oncogenic risk (HR) HPV types was tested for using the high-risk probe cocktail as previously described (Terry et al, 1994). Samples with optical densities more than 0.4 above background were considered as positive. Samples which were PCR/SHARP positive were assayed by semi-quantitative type-specific PCR for HPV16, 18, 31 and 33 (Cuzick et al, 1994).

*Received 20 January 1999*
*Revised 22 April 1999*
*Accepted 22 April 1999*

*Correspondence to:* J Cuzick

**Table 1** Cytology vs histology

| Cytology | Histology | | | | | | | |
|---|---|---|---|---|---|---|---|---|
| | Inadequate | Negative/ no biopsy | HPV/ borderline | CIN 1 | CIN 2 | CIN 3 | Adeno in situ | Total |
| Inadequate | 0 | 147 | 2 | 0 | 0 | 0 | 0 | 149 (5.0%) |
| Negative | 3 | 2636 | 19 | 9 | 1 | 5 | 0 | 2673 (89.4%) |
| Borderline | 4 | 59 | 23 | 7 | 1 | 2 | 0 | 96 (3.2%) |
| Mild | 0 | 10 | 8 | 4 | 4 | 3 | 0 | 29 (1.0%) |
| Moderate | 0 | 2 | 4 | 3 | 0 | 2 | 0 | 11 (0.4%) |
| Severe | 0 | 1 | 0 | 3 | 1 | 21 | 0 | 26 (0.9%) |
| Glandular atypia | 0 | 0 | 1 | 1 | 1 | 0 | 1 | 4 (0.1%) |
| Total | 7 (0.2%) | 2855 (95.6%) | 57 (1.9%) | 27 (0.9%) | 8 (0.3%) | 33 (1.1%) | 1 (0.03%) | 2988 (100%) |

Consensus PCR fragments from specimens positive by SHARP but negative for the four high-risk types by type-specific PCR were assessed visually by gel electrophoresis and those with band intensities equivalent to a control amplified in parallel from 10 fg of HPV 16 DNA were typed for HPV35, 51, 52, 56, 58 by restriction fragment polymorphism analysis as previously described (Londesborough et al, 1996). In this communication all samples identified by either of these two methods were scored as positive for the semi-quantitative HR-HPV assay. Women with a positive test for HR-HPV by SHARP/PCR or any cytological abnormality (borderline dyskaryosis or higher) were referred for colposcopy. Any apparent abnormality was biopsied and treated by loop excision as necessary. Histology was read independently by two pathologists who scored the results blindly without reference to cytological or other clinical information. There were very few discrepancies and when they did occur the final diagnosis was determined by consensus opinion.

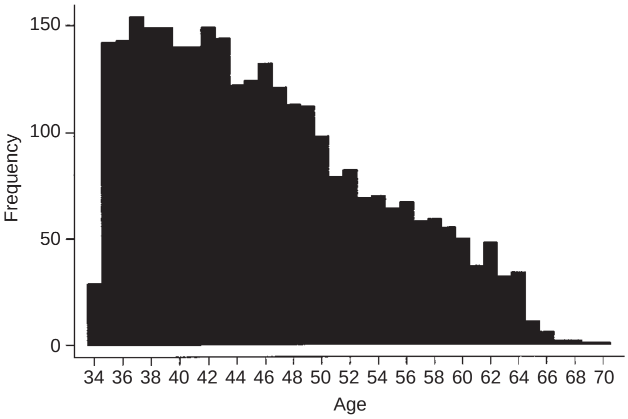

**Figure 1** Age distribution

### Statistical methods

The statistical methods used are mostly descriptive. The main results are presented by two-way contingency tables comparing cytology and HPV testing to the gold standard of histology. Relative sensitivity and specificity are determined by comparing the cases detected by each test to those positive for either test, but a few positives may have been missed if they were negative on both tests and thus were not referred for colposcopy. Positive predictive values are also compared. Referral for colposcopy was based on cytology and the Consensus PCR/SHARP assay. To evaluate the Hybrid Capture assay, all patients referred for colposcopy and a sample of negative controls were evaluated. Adjustments to the positivity rate were made to compensate for this sampling procedure. Trends of HPV positivity with age were evaluated by linear and quadratic logistic regression.

### RESULTS

A total of 3103 women were entered into the study. On review, 56 were found to be under the age of 35. Of these, 29 were aged 34 and were therefore included, but the 27 who were under the age of 34 were excluded. Another 20 were excluded because of previous treatment (15) or cytologic abnormality in the previous 3 years (five), and 68 were excluded because their cytology slide (19) or HPV sample (49) was damaged in transit. This left 2988 evaluable patients. The mean age was 46.0 years and the age distribution is shown in Figure 1. Some degree of dyskaryosis or glandular abnormality was found in 70 women (2.4%) and in another 96 cases (3.2%) borderline changes were reported (Table 1). Forty-two (1.4%) women had high-grade CIN on histology (CIN2 = eight, CIN3 = 33, adeno-in-situ = one). The sensitivity of cytology for high-grade CIN was 62% for moderate or severe dyskaryosis, or glandular atypia, 79% for any dyskaryosis and 86% when also including borderline changes (Table 1). The positive predictive value (PPV) was 63% for moderate/severe dyskaryosis, or glandular neoplasia, 24% for mild dyskaryosis and 3% for borderline changes. Overall, the PPV for mild dyskaryosis or worse was 47%, and for borderline or worse 22%.

Five per cent of smears were judged inadequate. All but eight of these were HPV-negative and no abnormalities were discovered as a result of repeat cytology. Eight patients with inadequate smears were HPV-positive. One of these patients developed a CIN3 lesion not seen at the initial colposcopy, but diagnosed 17 months later; the others were negative. The patient with CIN3 had a positive Hybrid Capture II Microtitre test and positive repeat PCR HPV test, but repeat cytology was negative.

Six per cent of the samples were HR-HPV-positive by the PCR/SHARP assay. Figure 2 shows that the rates were highest in the 35–39 age range and reached a nadir in the 45–49 age group, but increased again in the older age groups. A logistic regression with a linear and quadratic age term indicated that the variation was significant ($\chi^2 = 7.00$, 2 df, $P = 0.03$). The positivity rates in women without any histological evidence of abnormality was

© 1999 Cancer Research Campaign

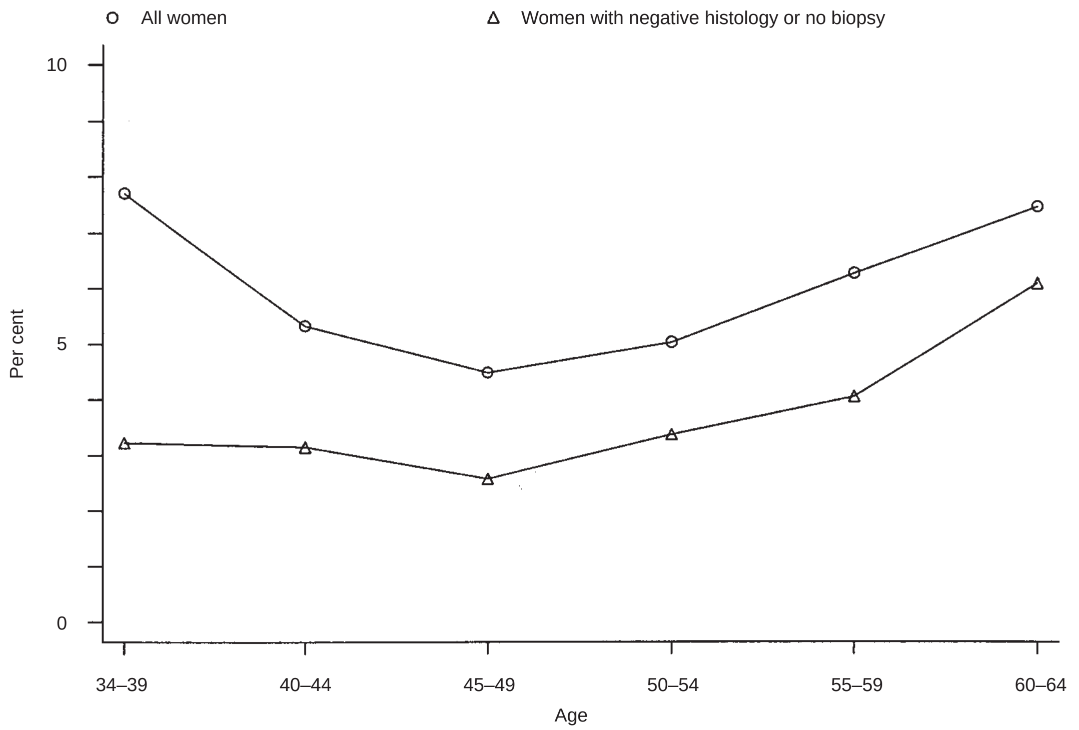

**Figure 2** HPV Positivity (SHARP PCR Assay) by age

**Table 2** Results of HR-HPV typing in PCR/SHARP-positive samples

| HPV type | Histology: Inadequate | Negative/ no biopsy | Borderline/ CIN 1 | CIN 2 | CIN 3 | Adeno in situ | Total |
|---|---|---|---|---|---|---|---|
| 16 | 0 | 3 | 6 | 2 | 9 | 1 | 21 |
| 18 | 0 | 1 | 0 | 1 | 3 | 0 | 5 |
| 31 | 0 | 3 | 5 | 1 | 9 | 0 | 18 |
| 33 | 0 | 1 | 1 | 0 | 0 | 0 | 2 |
| 35 | 0 | 2 | 0 | 0 | 0 | 0 | 2 |
| 51 | 1 | 1 | 1 | 0 | 0 | 0 | 3 |
| 52 | 1 | 1 | 6 | 0 | 0 | 0 | 8 |
| 56 | 0 | 0 | 2 | 0 | 1 | 0 | 3 |
| 58 | 0 | 2 | 1 | 0 | 3 | 0 | 6 |
| Other | 0 | 5 | 2 | 0 | 0 | 0 | 7 |
| Neg type | 2 | 75 | 29 | 2 | 2 | 0 | 110 |
| Total[a] | 3 | 93 | 50 | 4 | 26 | 1 | 177 |

Multi-types: HPV16 + 56, CIN3; HPV31 + 52, Bord/HPV; HPV16 + 52, no biopsy; HPV16 + 31, CIN1; HPV51 + 52, Inadequate; HPV11 + 40, Bord/HPV; HPV16 + 18 + 31, CIN2. [a]Multiple types counted more than once except in total.

3.4% (97/2876) and there was a slight increase with age, but this was not significant ($P = 0.32$, linear fit) (Figure 2). The sensitivity for CIN 3/adeno-in-situ was 79.4% and for all high-grade lesions 73.8%. The PPV of the PCR/SHARP test for high-grade CIN was 17.4%. Eleven high-grade lesions were negative for HPV and six were negative on cytology. Another ten only had borderline (three) or mild (seven) cytological abnormalities. The specificity and PPV test was improved when PCR/SHARP-positive samples were tested semi-quantitatively by type-specific HR-HPV, but at some loss of sensitivity (Table 2). Four high-grade lesions (two CIN2 and two CIN3) were counted as negative and this reduced the sensitivity from 74% to 64%. One hundred and six women who were PCR/SHARP-positive with less than CIN2/3 were found to have only low levels of HR-HPV and the PPV was improved substantially to 40% (27/67). Comparative results are shown in Table 3. This approach most closely parallels the approach taken in our previous work using type-specific PCR for HPV 16, 18, 31 and 33 (Cuzick et al, 1995), except that we have also added five other high-risk types (HPV 35, 51, 52, 56, 58). Of these, type 58 appears (on small numbers) to be most informative here as elsewhere (Huang et al, 1997). However, as before, types 16 and 31 were the most common and informative, both with PPVs above 50%.

The Hybrid Capture Test was evaluated restrospectively. For the first 1285 women, the Hybrid Capture Tube test (HC-I) was used

 © 1999 Cancer Research Campaign

**Table 3** Positivity rate (%) by histology for cytology and different HPV tests

| Assay | Histology | | | | | |
|---|---|---|---|---|---|---|
| | Negative/ no biopsy | Borderline/ CIN 1 (*n* = 84) | High grade (*n* = 42) | CIN 3 + (*n* = 34) | Total Positive | PPV for high grade |
| Cytology: moderate/severe | 0.1 | 14.3 | 61.9 | 70.6 | 1.4 | 63.4 |
| Cytology: mild or worse | 0.5 | 28.6 | 78.6 | 79.4 | 2.3 | 47.1 |
| Cytology: borderline or worse | 2.5 | 64.3 | 85.7 | 85.3 | 5.6 | 21.7 |
| SHARP (*n* = 2988) | 3.4 | 59.5 | 73.8 | 79.4 | 5.9 | 17.5 |
| Type specific | 0.5 | 25.0 | 64.3 | 73.5 | 2.0 | 40.3 |
| Hybrid Capture (Tubes) (*n* = 1285, but sampled) | 18.3[a] | 48.4 (*n* = 31) | 70.4 (*n* = 16) | 87.5 (*n* = 16) | 19.9[a] | 4.4[a] |
| Hybrid Capture Microtitre (1 pg) (*n* = 1703, but sampled) | 4.9[a] | 42.1 (*n* = 38) | 95.2 (*n* = 21) | 100 (*n* = 15) | 6.8[a] | 17.1[a] |
| Hybrid Capture Microtitre (2 pg) | 2.3[a] | 39.5 | 95.2 | 100 | 4.2[a] | 27.0[a] |
| Hybrid Capture Microtitre (4 pg) | 2.1[a] | 39.5 | 95.2 | 100 | 4.1[a] | 28.1[a] |

[a]Percentages adjusted for sampling scheme with a dacron swab for sample collection, whilst in the remaining 1703 patients the Hybrid Capture Microtitre format test (HC-II) was used with a conical cervical sampler brush. All cases (292) with non-negative cytology (166) or positive HR-HPV by PCR/SHARP (177) were assayed, except for six missing samples (three SHARP-positive, five cytology-positive, four of which were damaged in transit to the USA). A sample of completely negative controls was also assayed (62 for HC-I, 330 for HC-II). Adjustments were made for this sampling scheme.

The initial HC-I tests (carried out in 1995) had good sensitivity (87.5%), but the false positivity rate was unacceptably high (18.3%). More recently, we evaluated an additional 170 PCR/SHARP-negative samples by an improved HC-I test and found that there were only three false positives (1.8%). Two high-grade lesions (one CIN2, one CIN3) were both positive. Additional positive samples were not available to adequately re-evaluate sensitivity. The signal to noise ratio (S/N) for the 10 pg $ml^{-1}$ positive control in this improved HC-I assay was 6 compared to a S/N of 2 previously.

The HC-II microtitre test performed substantially better than the HC-I tube test. At the specified 1 pg $ml^{-1}$ cut-off the microtitre test had a 100% sensitivity for CIN3 and a 95.2% sensitivity for all high-grade lesions (20/21). The positivity rate in women with no evidence of CIN was reduced to 4.9%. In fact, all HPV-positive cases of CIN 2/3 had levels above 4 pg $ml^{-1}$, and if a higher cut-off threshold was used, a lower false positive rate was achieved (2.3% at 2 pg $ml^{-1}$, 2.1% at 4 pg $ml^{-1}$) without any loss of sensitivity. Since not all of the HC positives were colposcoped, some ‘false positives’ may be true positives, which would improve the specificity and PPV. Only two-thirds of the ‘false positive’ results for HC-II received colposcopy. At either of these thresholds the HC-II test had a better sensitivity and a better specificity than the HC-I test, the PCR/SHARP test, or cytology if borderline lesions are considered positive. The PPV for HC-II was 17%, 27% and 28%, at the 1, 2 and 4 pg $ml^{-1}$ cut-offs respectively.

The cytological diagnosis of the 11 PCR/SHARP-negative women with high-grade CIN showed five with high-grade cytology, four with mild dyskaryosis and two with borderline smears. Of these 11, ten had samples available for testing by HC-II and all of these were positive at above the 4 pg $ml^{-1}$ level.

### DISCUSSION

In this study the PCR/SHARP assay had a sensitivity similar to that of cytology but a lower specificity in predicting high-grade lesions. This is not unexpected since we showed previously that the identification of incident high-grade lesions is dependent on both the viral types (HR-HPV as detected by PCR/SHARP) and viral load (not measurable by PCR/SHARP). This is clearly shown when the PCR/SHARP-positive samples were further tested by semi-quantitative HR-HPV assay. All but three of the HPV-positive high-grade lesions were associated with types 16, 18, 31, or 33, which were the only types tested for in our previous study. The remaining three cases were all positive for HPV 58, suggesting this type should also be assayed in screening tests.

Overall, adding the PCR/SHARP assay to any abnormal cytology increased the yield of high-grade lesions by 17% from 36 to 42. This is substantially less than the 78% found in our previous study of young women (Cuzick et al, 1995). There are two possible explanations for this – either cytology performed substantially better and detected more of the high-grade lesions, or the HPV test performed worse, in that it failed to detect as many of the high-grade lesions missed by cytology. Since women who were negative on both tests were not referred for colposcopy, it is impossible to know for sure which of these is true. However, the very high correlation of high-grade cytology with high-grade histology suggests it may have been the former. The sensitivity of moderate or severe dyskaryosis for CIN 2/3 of 62% is well above that reported in the literature (Soutter et al, 1986; Walker et al, 1986; Hirschowitz et al, 1992; Jones et al, 1992) where typically more than half of high-grade lesions initially present with low-grade or borderline cytology (Kinney et al, 1998).

If HPV testing is to be used as an adjunct to cytology, it is important that the added sensitivity is achieved without greatly increasing the referral rate for colposcopy. Only persistent HPV infection is associated with high-grade CIN, and it may be appropriate to require two positive HPV tests 6 months apart before referring patients with negative cytology. Alternatively, specific predictors of persistence, such as high viral load, age, or HPV 16 may be adequate risk factors to justify referral after a single test.

© 1999 Cancer Research Campaign

Only three of the 96 women with borderline cytology had a high-grade CIN lesion and HPV testing may have a role in the triage of these women. One of these three was positive for HPV by PCR/SHARP, but all three were positive by Hybrid Capture. The PCR/SHARP-positive case also tested positive by HC-I, had HPV 31 and was found to have CIN3. The two PCR/SHARP-negative cases were both positive by HC-II (one CIN3, one CIN2).

A second objective was to evaluate the Hybrid Capture HPV test. This was done retrospectively by testing all women who were either PCR/SHARP- or cytology-positive (referred for colposcopy), and a sample of controls who were negative on both tests. Since none of the latter were referred for colposcopy it is possible that a few more cases might have been detected by the Hybrid Capture test. Evaluation was complicated by the fact that the Hybrid Capture test and collection device were changed in the middle of the study. The older prototype HC-I test had a slightly better sensitivity than the PCR/SHARP test, but a very poor specificity, leading to a 18% false positive rate, which is unacceptably high for a screening test. However, the new HC-II assay, which was used for about 60% of the study population, performed substantially better, with a sensitivity in excess of 95%, a false positive rate of 4.9% when used at the recommended cut-off level of 1 pg $ml^{-1}$. In this study, all the positive high-grade lesions had a value in excess of 4 pg $ml^{-1}$, and if a 2 pg $ml^{-1}$ cut-off was used, there was no loss of sensitivity and the false positive rate was reduced to 2.3%.

In summary, HPV testing for high-risk types may be a useful adjunct to cytology, especially if a quantitative assay is used. The new Hybrid Capture test (HC-II) is a strong candidate if the results reported here are reproducible. The detection rate of HC-II was better than cytology for high-grade CIN and the subjective evaluation and difficult borderline category is avoided. This higher sensitivity could lead to cost savings if it allows the screening interval to be safely lengthened and would more than compensate for the cost of the test (Cuzick and Sasieni, 1997). Another area where HPV testing may improve screening outcomes and save costs is for borderline smears. This could be achieved by referring HR-HPV-positive cases immediately and reducing surveillance in those who are HR-HPV-negative. However, a major concern for any screening test is the false positive rate. This was only 2.3% for HC-II when a higher cut-off point was used to determine positivity, and it may be possible to reduce this further by requiring a repeat HPV test when cytology is negative but HPV is positive. The role of HPV typing in management strategies requires further investigation.

### ACKNOWLEDGEMENTS

We gratefully acknowledge the expert work of Kit-Man Lui in data management. We also wish to thank Ian Phillips and the cytology department staff at the Hammersmith Hospital, the general practitioners, and practice nurses for their support throughout the study.

AT Lorincz and I Mielzynska are scientific staff at Digene Corporation, the company that developed the Hybrid Capture test.

### REFERENCES

Cox JT, Lorincz AT, Schiffman MH, Sherman ME, Cullen A and Kurman RJ (1995) Human papillomavirus testing by hybrid capture is useful in triaging women with a cytologic diagnosis of ASCUS. *Am J Obstet Gynecol* **172**: 946

Cuzick J and Sasieni P (1997) Estimates of the cost impact of introducing HPV testing into a cervical screening programme. In: *New Developments in Cervical Cancer Screening and Prevention*. Franco & Monsonego, pp 364–72. Blackwell Science Ltd. Oxford

Cuzick J, Terry G, Ho L, Hollingworth T and Anderson M (1994) Type-specific human papillomavirus DNA in abnormal smears as a predictor of high-grade cervical intraepithelial neoplasia. *Br J Cancer* **69**: 167–171

Cuzick J, Szarewski A, Terry G, Ho L, Hanby A, Maddox P, Anderson M, Kocjan G, Steele ST and Guillebaud J (1995) Human papillomavirus testing in primary cervical screening. *Lancet* **345**: 1533–1537

Ferenczy A, Franco E, Arseneau J, Wright TC and Richart RM (1996) Diagnostic performance of Hybrid Capture HPV DNA assay combined with liquid-based cytology. *Am J Obstet Gynecol* **175**: 651

Hatch KD, Schneider A and Abdel-Nour MW (1995) An evaluation of human papillomavirus testing for intermediate- and high-risk types as triage before colposcopy. *Am J Obstet Gynecol* **172**: 1150

Hildesheim A, Schiffman MH, Gravitt PE, Glass AG, Greer CE, Zhang T, Scott DR, Rush BB, Lawler P, Sherman ME, Kurman RJ and Manos MM (1994) Persistence of type-specific human papillomavirus infection among cytologically normal women. *J Infect Dis* **169**: 235–240

Hirschowitz L, Raffle AE, Mackenzie EF and Hughes AO (1992) Long term follow up of women with borderline cervical smear test results: effects of age and viral infection on progression to high grade dyskaryosis. *BMJ* **304**: 1209–1212

Ho GYF, Burk RD, Klein S, Kadish AS, Chang CJ, Palan P, Basu J, Tachezy R, Lewis R and Romney S (1995) Persistent genital human papillomavirus infection as a risk factor for persistent cervical dysplasia. *J Natl Cancer Inst* **87**: 1365–1371

Huang S, Afonina I, Miller BA and Beckmann AM (1997) Human papillomavirus types 52 and 58 are prevalent in cervical cancers from Chinese women. *Int J Cancer* **70**: 408–411

Jones MH, Jenkins D, Cuzick J, Wolfendale MR, Jones JJ, Balogun-Lynch C, Usherwood M McD and Singer A (1992) Mild cervical dyskaryosis: safety of cytological surveillance. *Lancet* **339**: 1440–1443

Kinney WK, Manos MM, Hurley LB and Ransley JE (1998) Where's the high grade cervical neoplasia? The importance of minimally abnormal Papanicolaou diagnoses. *Obstet Gynecol* **91**: 973–976

Londesborough P, Ho L, Terry G, Cuzick J, Wheeler C and Singer A (1996) Human papillomavirus genotype as a predictor of persistence and development of high-grade lesions in women with minor cervical abnormalities. *Int J Cancer* **69**: 364–368

Soutter WP, Wisdom S, Brough AK and Monaghan JM (1986) Should patients with mild atypia in a cervical smear be referred for colposcopy? *Br J Obstet Gynaecol* **93**: 70–74

Terry G, Ho L, Szarewski A and Cuzick J (1994) Semiautomated detection of human papillomavirus DNA of high and low oncogenic potential in cervical smears. *Clin Chem* **40**: 1890–1892

Walboomers JMM, de Roda Husman AM, Snijders PJF, Stel HV, Risse EKJ, Helmerhorst TJM, Voohorst FJ and Meijer CJLM (1995) Human papillomavirus in false negative archival cervical smears: implications for screening for cervical cancer. *J Clin Pathol* **48**: 728–732

Walker EM, Dodgson J and Duncan ID (1986) Does mild atypia on a cervical smear warrant further investigation? *Lancet* **ii**: 672–673

 © 1999 Cancer Research Campaign
